# Factors Associated with Complicated Appendicitis: View from a Low-middle Income Country

**DOI:** 10.7759/cureus.4765

**Published:** 2019-05-28

**Authors:** Muhammad Sohaib Khan, Muhammad Tayyab H Siddiqui, Noman Shahzad, Aleezay Haider, Mustafa Belal Hafeez Chaudhry, Rehman Alvi

**Affiliations:** 1 Surgery, Aga Khan University, Karachi, PAK; 2 General Surgery, East Kent Hospitals University National Health Service Foundation Trust, Margate, GBR; 3 Radiology, Aga Khan University, Karachi, PAK

**Keywords:** complicated appendicitis, perforated appendicitis, appendicoliths

## Abstract

Introduction

Factors associated with complicated appendicitis have been inconsistently identified. Moreover, studies are lacking from low and low-middle countries where access to surgical care is limited. Our objective was to identify factors predicting complicated appendicitis as diagnosed intraoperatively in a low-middle income country hospital.

Methodology

Retrospective case-control study of patients who underwent laparoscopic appendectomy from 01/2008 to 12/2015 was completed. Based on intraoperative diagnosis of complicated appendicitis, patients were divided into two groups; those with complicated appendicitis (CA) and those who had non-complicated appendicitis (NCA). CT scans were further reviewed to identify presence of appendicolith.

Result

Of the 442 patients included, 88 (20%) patients were in the CA group while 354 (80%) patients were in the NCA group. Patients in the CA group were older [CA vs. NCA: 34.6 ± 14 vs. 30.4 ± 11.5; p-value < 0.001], had symptoms for longer duration [CA vs. NCA: 2 ± 1.2 vs. 1.5 ± 0.8; p-value: 0.001] and had a greater proportion of patients with appendicoliths [CA vs. NCA: 37 (42%) vs. 84 (23.7%); p-value: 0.001]. On multivariable regression analysis, patients with complicated appendicitis had greater odds of having appendicoliths (OR: 2.4, 95% CI: 1.4-4.07; p-value < 0.001) and symptoms for a longer duration (OR: 1.57, 95% CI: 1.25-1.97; p-value < 0.001).

Conclusion

Patients with complicated appendicitis had greater odds of having appendicoliths and symptoms for a longer duration. Further studies are warranted in low and low-middle income countries to gauge the impact delay in presentation and intervention has on appendicitis and its outcomes.

## Introduction

Acute appendicitis is one of the common indications for emergency surgery globally [1]. Incidence in recently industrialized countries has been reported to be over 200 per 100,000 person years. One-third of patients who develop appendicitis are found to have complicated appendicitis at presentation which includes appendiceal perforation, gangrene and abscess formation [2,3]. Up to 35% of patients who undergo appendectomy for complicated appendicitis are reported to have post operative complications such as surgical site infections, ileus and bowel obstructions [3]. Moreover, variable outcomes have been reported from different parts of the world. In low and low-middle income countries, perioperative mortality is reported to be range from 2.4 per 1000 appendectomies in South Asia to 54 per 1000 appendectomies in Central sub-Saharan Africa [4].

Perforated and non-perforated appendicitis have different incidence rates over time leading to the hypothesis that the underlying pathophysiology may be different [5,6]. Factors that predict complicated appendicitis have been inconsistently identified. Studies have identified a delay in surgery as a factor associated with increased risk of perforation and postoperative complications [7-9]. This is particularly important in low-middle and low income countries where access to surgical care is limited [4]. However a recent meta-analysis contradicted the importance of delay in surgery [10]. Factors like male gender, appendicoliths and diabetes mellitus (DM) have also been linked with appendiceal perforation [11,12]. Other studies have refuted these associations [13,14]. Moreover, these studies were based on CT imaging which has a low sensitivity of identifying complicated appendicitis [15,16]. Intraoperative diagnosis is more efficient for identification of complicated appendicitis.

Our objective in this study was to identify factors associated with complicated appendicitis as this is particularly important in low and low-middle income countries where access to surgical care is limited.

## Materials and methods

A retrospective case-control study of patients who underwent laparoscopic appendectomy from January 2008 to December 2015 was completed at the Aga Khan University Hospital Karachi, Pakistan. Patients who were clinically suspected of having appendicitis, received a pre-operative abdominal CT scan and underwent laparoscopic appendectomy were included in the study. Approval from institutional ethics review committee was obtained prior to start of the study.

Data of potentially confounding variables namely gender, age, comorbidities and symptom duration were collected. CT scans were rereviewed to identify presence of appendicoliths. Diagnosis of complicated appendicitis (CA) and non-complicated appendicitis (NCA) was based on the operative findings. Perforated or gangrenous appendicitis with or without an abscess were termed as complicated appendicitis.

Categorical variables were reported as frequencies with percentages. Continuous variables were expressed as mean ± standard deviation. Chi-square and Independent Samples T-Test were performed. All variables with p-values less than 0.2 were included in the multivariate regression analysis which was used to calculate odds ratios (OR) and 95% confidence intervals (CI). The statistical analysis was performed using the Statistical Software Package SPSS 20.0 (SPSS, Chicago, IL).

## Results

In total, 442 patients were included in the study. Of these, 88 (20%) patients were in the CA group while 354 (80%) patients were in the NCA group. While the gender distribution between the two groups was similar, the patients in the CA group were significantly older than those in the NCA group [CA vs. NCA: 34.6 ± 14 vs. 30.4 ± 11.5; p-value < 0.001] (Table 1).

**Table 1:** Characteristics of patients with complicated and non-complicated appendicitis. CA: Complicated appendicitis; NCA: Non-complicated appendicitis.

	CA (n = 88)	NCA (n = 354)	p-value
Mean Age ± SD (years)	34.6 ± 14	30.4 ± 11.5	<0.001
Females (%)	29 (33)	105 (29.7)	0.5

On univariate analysis, patients in the CA group had symptoms for a significantly longer duration [CA vs. NCA: 2 ± 1.2 vs. 1.5 ± 0.8; p-value: 0.001] and had a greater proportion of patients with appendicoliths [CA vs. NCA: 37 (42%) vs. 84 (23.7%); p-value: 0.001] (Table 2).

**Table 2:** Univariate analysis of predictive factors and complicated appendicitis. CA: Complicated appendicitis; NCA: Non-complicated appendicitis.

Variables			CA (n = 88)	NCA (n = 354)	p-value
Diabetes Mellitus	Present		6 (6.8%)	18 (5.1%)	0.5
	Not Present		82 (93.2%)	336 (94.9%)	
Mean Symptom Duration ± SD (Days)			2 ± 1.2	1.5 ± 0.8	<0.001
Appendicolith		Present	37 (42%)	84 (23.7%)	0.001
		Not Present	51 (58%)	270 (76.3%)	
Total Leukocyte Count		>12000	37 (42%)	146 (41.2%)	0.89
		<12000	51 (58%)	208 (58.8%)	

On multivariable regression analysis, odds of having appendicoliths was 2.4 times greater for patients with complicated appendicitis (OR: 2.4, 95% CI: 1.4-4.07; p-value < 0.001). Additionally, odds of complicated appendicitis increased 1.5 times with each additional day from the onset of symptoms (OR: 1.57, 95% CI: 1.25-1.97; p-value < 0.001) (Table 3).

**Table 3:** Multivariable logistic regression analysis of factors associated with complicated appendicitis.

Variables	Odds Ratio (OR)	p-value	95% Confidence Interval (CI)	
Age	1.02	0.008	1.007	1.04
Symptom Duration	1.57	<0.001	1.25	1.97
Appendicolith	2.4	0.001	1.46	4.07

## Discussion

Patients diagnosed with complicated appendicitis intraoperatively had greater odds of having appendicoliths and symptoms for a longer duration. Up to 30% of patients with appendicitis are reported to have complicated appendicitis [17]. Surgical management of these patients is associated with postoperative complications in up to 35% of patients. These complications include surgical site infections, bowel obstruction, ileus and cardiac complications [3]. Given the large burden and associated postoperative complications, identifying the associated factors is of great clinical relevance. Duration of symptoms, potentially a modifiable risk factor, is influenced by barriers that determine access to surgical care [18]. Appendicoliths, a non-modifiable risk factor, is estimated to be present in up to 30% of asymptomatic population [19]. Both of these factors have been shown to increase the risk of complicated appendicitis in other recent studies as well [11,20,21].

Delays in surgery after onset of symptoms and after admission have both been associated with complicated appendicitis [9,21]. Papandria et al. in an analysis of 683,590 patients with appendicitis found that the odds of appendiceal perforation on the 8th day after admission increased to 4.7 time for adults and 15.4 times for children [9]. In a prospective study of 266 patients, Saar et al. found an incremental rise in severity and proportion of appendiceal perforation with each 12 hour delay after symptom onset [21]. In low-middle income countries delayed presentation for surgical conditions is common and multifactorial [18]. As much as 97% of the population of South Asia and Sub-Saharan Africa is estimated to lack access to timely essential surgical care when needed [22]. The impact this lack of access has is reflected in variability of perioperative mortality in appendectomies performed in low and middle income countries. Perioperative mortality ranges from 2.4 per 1000 appendectomies in South Asia to 54 per 1000 appendectomies in Central Sub-Saharan Africa [4]. However, studies that can determine the impact of delayed presentation on complicated appendicitis and its outcomes from low and low-middle income countries are lacking. This is one of the first studies from low-middle income countries that has looked into this relationship and has shown the odds of complicated appendicitis increased by 1.5 times for each day after the onset of symptoms.

Appendicoliths, also known as fecaliths, have historically been associated with appendicitis [23]. However, they can also be asymptomatic [24,25]. In light of recently published evidence, appendicoliths have been shown to exacerbate appendicitis [11,20,26]. Ishiyama et al. found an association of appendicoliths that were large and present at the base of the appendix with appendiceal perforation and gangrene [11]. Imran et al. described the increased odds of perforated appendicitis with greater symptom duration and the presence of an appendicolith [20]. The odds of complicated appendicitis in this study were increased by 2.4 times for those who had appendicoliths.

It is uncertain which modality best detects appendiceal perforations. Multiple CT scan findings are considered useful for diagnosis of complicated appendicitis [12]. However, recent studies have shown that CT scan findings lack sensitivity in detecting appendiceal perforations [16,27]. Intraoperative assessment may also overestimate appendiceal perforations by 40% [20]. While histopathological diagnosis is regarded as the gold standard, the final report takes many days to become available. For prevention of any subsequent complication, interventions have to take place in immediate perioperative period. Thus, for the timely diagnosis of appendiceal perforation intraoperative assessment is the most efficient method until other faster modalities become available.

This study is one of the first studies from low-middle income countries regarding factors associated with complicated appendicitis. It determines the impact delay in presentation has on outcomes and stresses the importance of timely diagnosis and urgent intervention. However, there are limitations. Our center is one of the largest urban centers in the country with 24-hour availability of radiological imaging such as CT scans. Thus, the impact of inaccessibility to surgical care and lack of radiological imaging as seen in rural settings is not reflected. In-patient delays have not been accounted for, although their impact at our center is likely to be minimal as almost all patients undergo surgery within 24 hours of admission.

## Conclusions

Patients with complicated appendicitis had greater odds of having appendicoliths and symptoms for a longer duration. Since access to surgical care is limited in low and low-middle income countries, further studies are warranted to gauge the impact delay in presentation has on appendicitis and its outcomes. Presence of an appendicolith also has the potential to be a marker for complicated appendicitis and can be used as a guide for timing the surgical intervention. This study provides basis for future interventional studies that aim to address the risks posed by complicated appendicitis.
